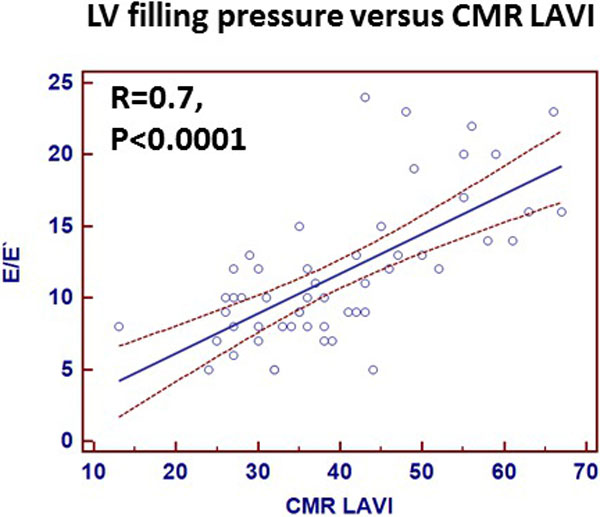# Incremental value of CMR over echocardiography for assessment of LV diastolic function

**DOI:** 10.1186/1532-429X-16-S1-P275

**Published:** 2014-01-16

**Authors:** Shashi Ranjan, Desmond Hardman, Prasad Challa, LS Bruno Jesuthasan, Mark Hansen, Arun Dahiya

**Affiliations:** 1 cardiology, Logan Hospital, Brisbane, Queensland, Australia; 2 Radiology, Logan Hospital, Brisbane, Queensland, Australia; 3 Radiology, Q Scan, Brisbane, Queensland, Australia; 4 Cardiology, Griffith university, Brisbane, Queensland, Australia

## Background

Incidence of heart failure with preserved ejection fraction is increasing and putting the emphases on comprehensive evaluation of diastolic function. Furthermore, diastolic dysfunction even in the absence of heart failure has been shown to have prognostic significance. American Society of Echocardiography and European Association of Echocardiography (ASE/EAE) 2-level decision tree using multi-parametric approach is comprehensive, however discrepancy between the LV filling pressures and LA volume index can lead to discordance and inaccurate assessment of diastolic function. Incremental value of Cardiac Magnetic Resonance (CMR) to Echocardiography in assessment of diastolic function remains unknown. Aim:To assess incremental role of CMR derived LA volume index in classifying diastolic function by Echocardiography.

## Methods

We studied consecutive patients undergoing both Echocardiography and Cardiac MRI at our institution. All patients underwent comprehensive diastolic function evaluation by echocardiography. CMR SSFP(steady state free precession) cine images were used to evaluate Left Atrial volume index (LAVI) (using area length method) and LA ejection Fraction (LAEF). Echo derived LV filling pressures were correlated with Echo and CMR derived LAVI and LAEF. Impact of substituting CMR derived LAVI for ECHO derived LAVI on diastolic function classification was also evaluated.

## Results

52 patients underwent CMR and ECHO (32 males, Age 51 ± 18 years, BSA 2.0 ± 0.25 m2). Mean LVEF and LVMI ( LV mass index) were 58 ± 5% and 95 ± 30 g/m2 respectively. Echo derived E/E' (12 ± 5) was used to evaluate LV filling pressures. CMR derived LAEF was 37 ± 9%. 12 patients had elevated filling pressures (E/e≥ 15), only 50% of these patient had elevated echo derived LAVI but all 12 patients had elevated CMR derived LAVI. ECHO derived LAVI (34 ± 14 ml/m2) underestimated CMR derived LAVI (40 ± 12 ml/m2). Substituting CMR derived LAVI for Echo derived LAVI improved the concordance between elevated filling pressures and LAVI reclassifying all patients with elevated filling pressures into pseudo normal diastolic function. Compared to echo derived LAVI, CMR derived LAVI had superior correlation to Echo derived LV filling pressures (Fig). Furthermore LAEF had inverse correlation with LV filling pressures(R = -0.7).

## Conclusions

CMR derived LAVI has superior correlation to Echo derived LV filling pressures and can be of incremental value over Echocardiography in assessing diastolic function. These findings are likely attributable to superior spatial resolution of CMR and atrial foreshortening by echocardiography.

## Funding

No funding support.

**Figure 1:**
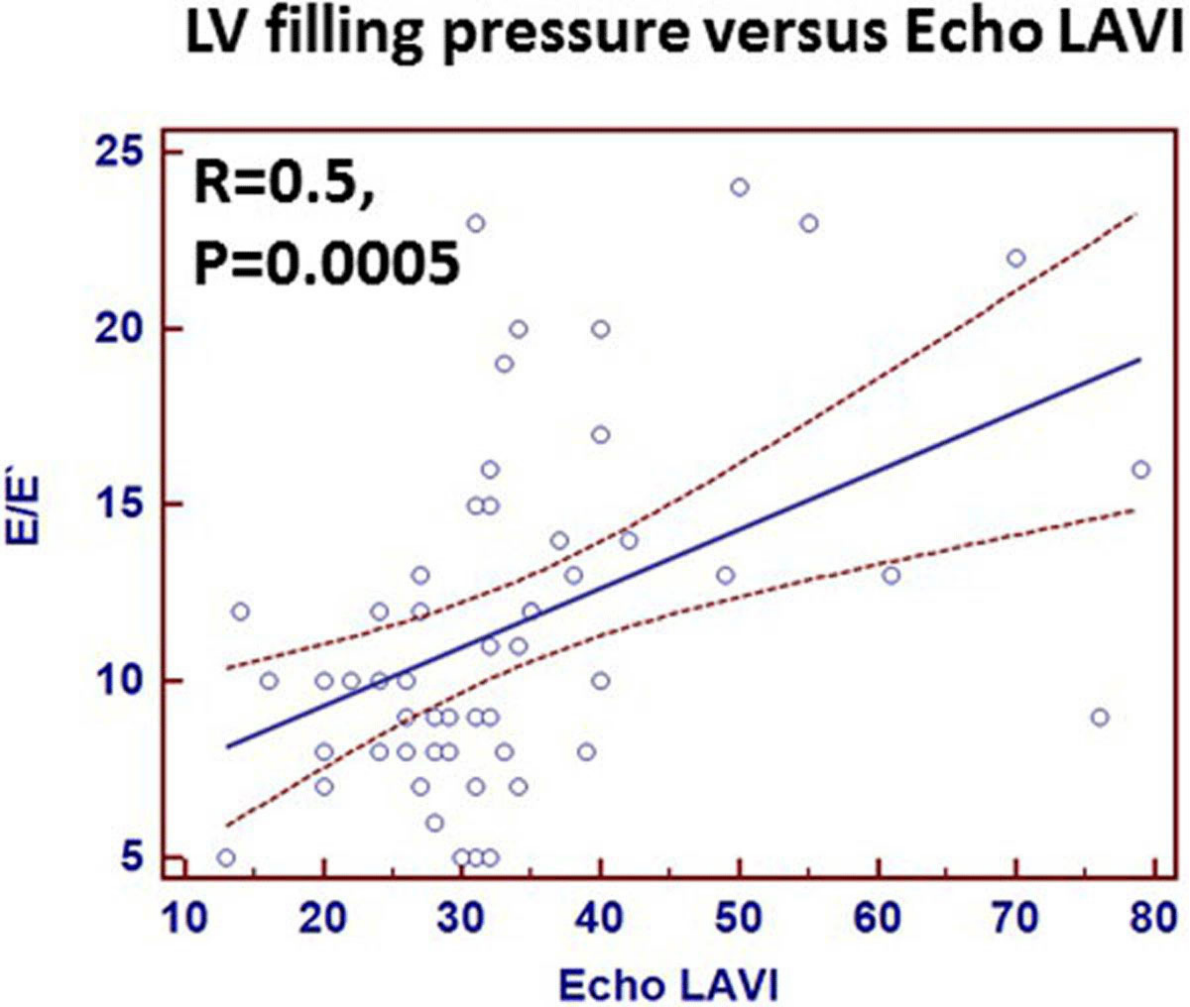


**Figure 2:**